# Surgery for the acute dissections of the ascending aorta and the arch

**DOI:** 10.1186/1749-8090-8-S1-O23

**Published:** 2013-09-11

**Authors:** M Kocica, D Cvetkovic, Lj Soskic, F Vucicevic, M Grujic, V Jovicic, A Mikic, M Matkovic, M Micic, M Ristic

**Affiliations:** 1 Clinic for Cardiac Surgery, UC Clinical Centre of Serbia, Belgrade, Serbia

## Background

Acute ascending aortic dissection is life-threatening condition that requires emergency surgery. When left untreated, about 33% of patients die within the first 24 hours, and 50% die within 48 hours. The aim of our study was to evaluate the clinical characteristics, management, and outcomes of patients with acute type A aortic dissection during the 5 years period.

## Methods

From January 2008 to December 2012, 227 patients (168 men, 59 women) aged 12 to 84 years (mean 58) underwent emergency operation for type A aortic dissection. Mean admission-to-table time was 2.3 (1-19) hours. Cannulation was accomplished in 211(92.9%) patients through the femoral artery. Supracommissural replacement of the ascending aorta was applied to 146 (64.3%) patients. In 52 patients (22.9%), the aortic valve was replaced either independently (11 pts, 4.8%) or by means of a composite graft (35 pts, 15.4%). “Open distal anastomosis” strategy was applied in 63 patients (27.8%) with isolated replacement of the ascending aorta, 47 pts (20.7%) with transverse arch, and 10 pts (4.4%) with total arch replacement, 3 (1.3%) of whom had an “elephant trunk”graft extension.

## Results

Overall early mortality was 23.3% (53 pts): operative 7.5% (17 pts) and hospital 15.9% (36 pts). The early major postoperative complications were: low cardiac output syndrome 28 pts (12.3%), hemorrhage 24 pts (10.5%), focal neurological deficit 26 pts (11.4%), coma 16 pts (7.0%) and acute renal failure 12 pts (5.3%).

## Conclusions

During this period of time, we have adopted a strategy of "the earliest possible surgery", reducing preoperative diagnostic algorithm on carefully clinical examination and the least possible number of imaging tools. The more successful tactics of aortic dissection treatment should focus on: earlier clinical suspicion and diagnosis decrease in "onset-to-admission" time, improvements in surgical strategy/ technique and establishment of National aortic dissection registry.

